# Editorial: Oxidative Stress, Antioxidants, Transcription Factors, and Assimilation of Signal Transduction Pathways in Obesity-Related Disorders

**DOI:** 10.3389/fphar.2021.759468

**Published:** 2021-09-07

**Authors:** David E. Stec, Barbara Wegiel, Terry D. Hinds Jr

**Affiliations:** ^1^ Department of Physiology and Biophysics, Cardiorenal and Metabolic Diseases Research Center, University of Mississippi Medical Center, Jackson, MS, United States; ^2^ Department of Surgery, Division of Surgical Oncology, Cancer Research Institute, Beth Israel Deaconess Medical Center and Harvard Medical School, Boston, MA, United States; ^3^ Department of Pharmacology and Nutritional Sciences, Barnstable Brown Diabetes Center, Markey Cancer Center, University of Kentucky, Lexington, KY, United States

**Keywords:** obesity, insulin resistance, therapeutics, bilirubin, reactive oxygen species, inflammation

Obesity and its comorbidities such as fatty liver disease, type II diabetes, and cardiovascular disease can cause an onset of complications, worsened by the accompanying oxidative stress and deleterious signaling mechanisms, leading to irreversible damages. For instance, long-term fat accumulation in the liver may induce oxidative stress, sterile inflammation, and ultimately fibrosis, currently considered as an irreversible event. Oxidative stress occurs with an imbalance of heightened free radicals and reduced antioxidant pool in the body. Reactive oxygen species (ROS) are free oxygen-containing molecules that quickly react with other molecules within the cell leading to tissue damage. This is particularly evident in patients with obesity and metabolic disorders. Nicotinamide adenine dinucleotide phosphate (NADPH) oxidases and the mitochondrial oxidative chain complexes generate the majority of ROS in obesity and the metabolic syndrome, and this can contribute to renal dysfunction [reviewed in (Lee and Jose)]. To combat this complication, antuioxidants are the body’s natural defense against ROS. They can be part of an extensive, integrated antioxidant defense system (*i.e.,* glutathione, bilirubin) or derived from vitamins or nutritional sources such as vegetables, fruits, nuts, and seeds. Antioxidants have been proposed as potential therapies for obesity and its associated pathologies. As part of this collection of articles, Tun et al. reviewed the role of oxidative stress and the potential therapeutic role for several antioxidants in obesity and its related comorbidities (Tun et al.).

Adipose tissue is not simply a storage receptacle for excess fats that occur in obesity, but rather it is a complex endocrine organ. Adipose tissue releases hormones such as leptin and adiponectin as well as several cytokines. Hence, these are referred to as adipokines, and some are beneficial for metabolic disease (adiponectin), and others can be disadvantageous and induce inflammation (TNFα). Obesity alters the release of adipokines contributing to metabolic dysfunction and other phenotypes such as altered bone metabolism. Adiponectin levels are lower in the obese patients and may affect cementoblasts’ mineralization rate, impacting periodontal tissue homeostasis and orthodontic treatment (Yong et al.). Excessive fructose consumption results in metabolic dysfunction and contributes to insulin resistance, vascular dysfunction, and hypertension. The hydrogen sulfide (H_2_S) system is composed of enzymatic and non-enzymatic pathways capable of scavenging ROS in the cytosol and the mitochondria. Pavlovskiy et al. used H_2_S donors to reduce fructose-induced gastric injury and demonstrated that they work in part by decreasing oxidative damage caused by fructose (Pavlovskiy et al.). Retinopathy is a common complication of diabetes. Hyperglycemia-induced ROS and inflammation are contributing factors to this condition. Yang et al. provided evidence that synthetic relaxin-3 (H3 relaxin) attenuated high glucose-induced nod-like receptor protein 3 (NLRP3) inflammasome activation in streptozotocin (STZ)-induced diabetic rats (Yang et al.).

Epigenetic genome modification is a tool for regulating gene expression without changes in the genomic sequence itself. Sirtuin-6 (Sirt6) is a histone deacetylase inhibitor that controls telomere length, DNA repair, and other cellular processes. Raj et al. highlighted the emerging role of Sirt6 in the prevention of metabolic diseases and discussed the potential therapeutic use of Sirt6 modulators (Raj et al.). Histone deacetylase (HDAC) inhibitors improved fatty-acid induced insulin resistance in mice fed high fat and high fructose diets through enhanced fatty acid oxidation and improved mitochondrial function (Lee et al.).

The body produces a potent antioxidant, bilirubin, a product of heme catabolism by the enzyme, heme oxygenase-1 (HO-1), which combats ROS and oxidative stress. Mildly elevated plasma bilirubin levels correlate with reduced risks of cardiovascular and metabolic diseases (Hinds and Stec, 2018; Weaver et al., 2018; Hinds and Stec, 2019). Primary cells (podocytes and aortic endothelial cells) isolated from hyperbilirubinemic Gunn rats exhibited significantly higher viability in response to palmitic acid or angiotensin II treatment (Bianco et al.). Gunn rats also displayed sex-dependent decreases in fat mass, elevated hepatic β-oxidation, and mitochondrial function (Vidimce et al.). These studies were supported by Hana et al*.*, who showed in this series that bilirubin treatment inhibited lipid accumulation in both skeletal muscle and liver cells and that this was dependent upon the glucose level (Hana et al.). While bilirubin is a well-known antioxidant, it might also have a hormonal function as a ligand [reviewed in (Creeden et al., 2020)] for the nuclear receptor peroxisome proliferator-activated receptor-α (PPARα) (Stec et al., 2016; Gordon et al., 2019; Gordon et al., 2020; Gordon et al., 2021). Hinds et al. utilized water-soluble bilirubin nanoparticles to treat obesity and its comorbidity, fatty liver disease. They showed that the bilirubin nanoparticles reduced dietary-induced obesity and hepatic steatosis via the induction of PPARα transactivation, accelerating β-oxidation and the utilization of fat in the liver, raising plasma levels of the ketone β-hydroxybutyrate, a known hepatic fatty acid oxidation marker (Hinds et al.).

Nature antioxidant remedies have been used for centuries in traditional herbal medicines like fenugreek, which treat a wide variety of ailments (Stec and Hinds, 2020). Yang et al. used 4-hydroxyisoleucine, the active component of fenugreek, to prevent dietary-induced obesity inflammatory response via modification of macrophage phenotype in the adipose and liver tissues (Yang et al.). Natural products exhibit antioxidant actions through induction of pathways such as HO-1 or nuclear factor erythroid 2-related factor 2 (Nrf2) (Stec and Hinds, 2020). Kaempferol is a flavonoid compound and antioxidant found in fruits and vegetables. Yao et al. demonstrated that the vascular protective effects of kaempferol are due to its antioxidant actions via induction of Nrf2/HO-1 (Yao et al.). Bauhiniastatin-1, the active component derived from the flowering plant Bauhinia purpurea, decreased high-fat diet-induced weight gain, adiposity, and insulin resistance in rats via modulation of peroxisome proliferator-activated receptor- γ (PPARγ) and AMP-activated protein kinase (AMPK) (Karunakaran et al.).

ROS and inflammation have active roles in obesity and its related pathologies. Antioxidant treatment with scavengers of ROS, cellular and mitochondrial ROS production inhibitors, or natural products that activate endogenous antioxidant pathways are potential therapies to mitigate obesity and metabolic diseases. Contributing articles to this research topic have highlighted how antioxidants may impact specific signaling pathways and transcriptional programs. Either through treatment with natural products or endogenous metabolites and hormones such as bilirubin and relaxin, targeting ROS and its associated transcription factors and signal transduction pathways offers a promise for therapy to combat obesity and its associated metabolic disorders.
